# Incidental Diagnosis of Primary Pleural Liposarcoma in a COVID-19-Positive Patient

**DOI:** 10.7759/cureus.42207

**Published:** 2023-07-20

**Authors:** Khoula Al Harrasi, Adil H Al-Kindi, Abdullah Al Lawati, Fatma Al Hosni, Asma Al Shezawi

**Affiliations:** 1 General Surgery, Sohar Hospital, Sohar, OMN; 2 Cardiothoracic Surgery, Sultan Qaboos University Hospital, Muscat, OMN; 3 College of Medicine and Health Sciences, Sultan Qaboos University, Muscat, OMN; 4 Radiology, Sohar Hospital, Sohar, OMN

**Keywords:** mediastinal shift, thoracotomy, myxoid liposarcoma, primary liposarcoma, covid-19

## Abstract

A few cases of primary liposarcoma of pleura have been reported worldwide. We report a young gentleman who was admitted with what was initially thought as coronavirus disease 2019 (COVID-19)-related pulmonary symptoms. His chest CT showed a large pleural effusion causing a mediastinal shift and compressing vital structures. He did not respond to conservative drainage intervention and subsequently underwent a left thoracotomy for his worsening clinical picture. He was found to have a large left pleural mass that was incompletely resected. Histopathology examination showed low-grade soft tissue sarcoma with lipogenic differentiation suggestive of myxoid liposarcoma. He was subsequently given adjuvant chemotherapy but succumbed and died because of the progression of the disease.

## Introduction

Liposarcoma is a malignant mesenchymal tumor that accounts for 15% to 20% of all malignant mesenchymal tumors [1]. Liposarcoma most commonly affects the lower extremities or the retroperitoneum [2]. An intrathoracic origin is unusual, and most liposarcomas in the thoracic region develop in the mediastinum, accounting for 2.7% of all occurrences [3,4]. The most prevalent kind of primary pleural liposarcoma (PPL) is the myxoid variety [5]. A few cases of primary liposarcoma of pleura have been reported worldwide. To our knowledge, this is the first reported case from the Sultanate of Oman.

## Case presentation

**Figure 1:**
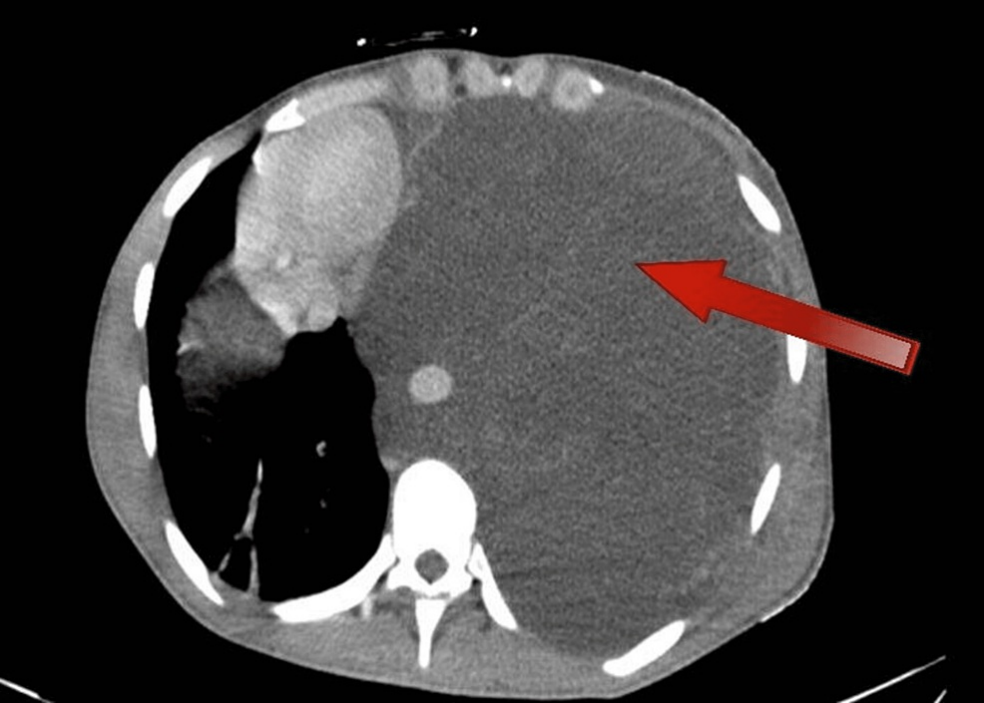
The left hemothorax mass causing mediastinal shift

**Figure 2:**
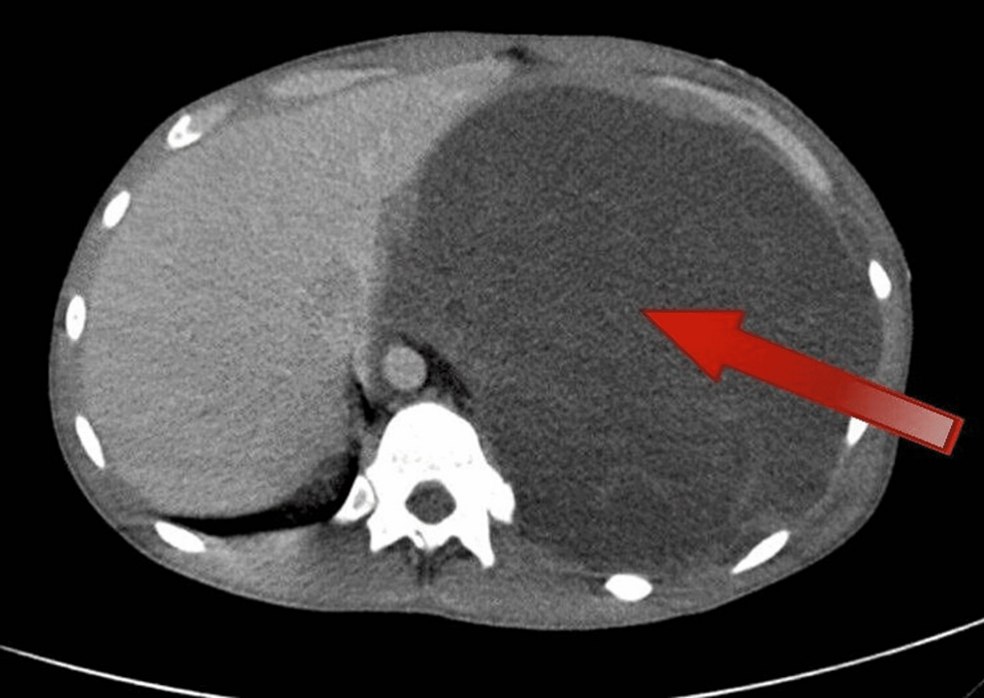
The left mass compressing on the left hemidiaphragm down to the abdomen

**Figure 3:**
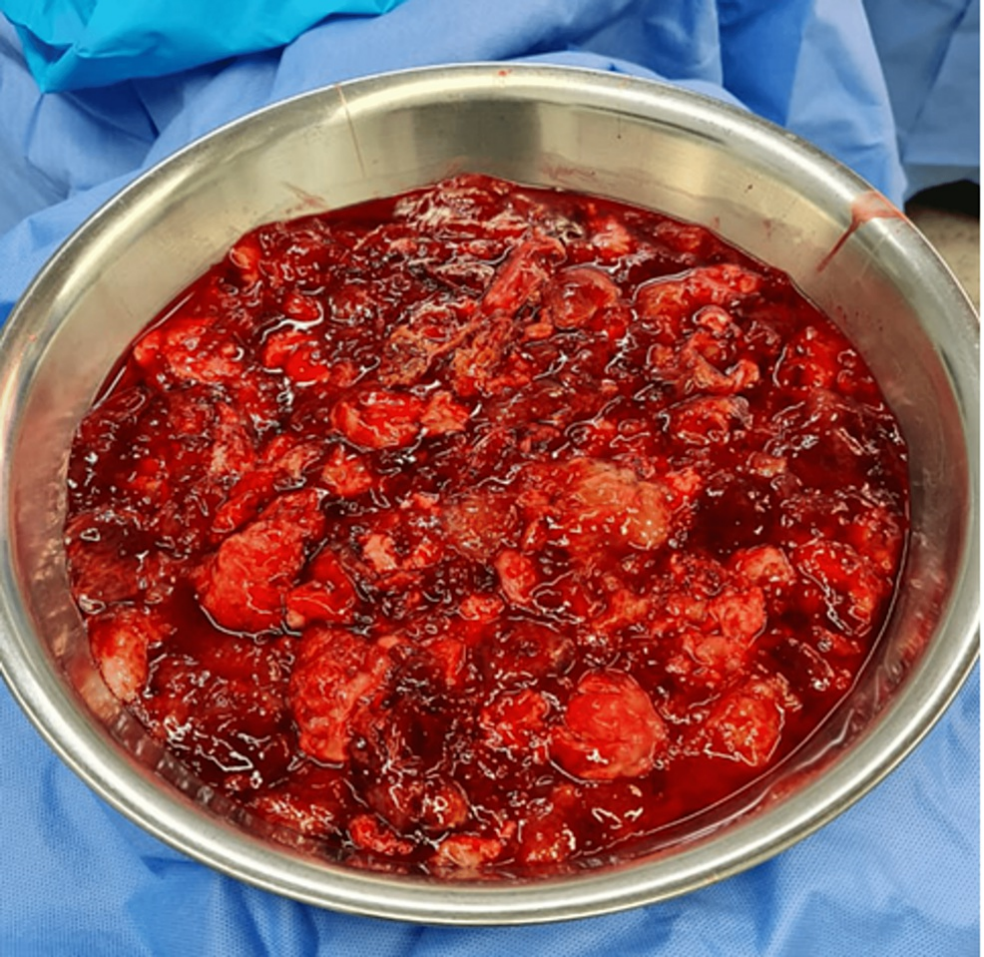
Fragments of the mass, which was gelatinous with no clear capsule

A 24-year-old male presented to the emergency department at our hospital with a two-week history of shortness of breath (SOB) and left-sided pleuritic chest pain. He had an expectorant cough but no hemoptysis, fever, chills, or rigor. He was a non-smoker with no significant past medical history, weight loss, or identifiable occupational exposure. He tested positive for coronavirus disease 2019 (COVID-19), and his chest X-ray (CXR) showed a large left-sided opacity with a right-sided mediastinal shift suggestive of a massive pleural effusion. A computed tomography (CT) showed a huge left-sided hemothorax/pleural effusion with numerous enhancing septations that depressed the hemidiaphragm and caused a significant rightwards mediastinal shift. In addition, the heart and great vessels were compressed, and the left lung was consolidated with some sparing of the left upper lobe, as shown in Figures 1, 2. A trial of intercostal chest drain (ICD) placement was done on the left side, as left-sided hemothorax was suspected; however, there was minimal output. The pleural fluid was negative for malignancy. The patient was referred to the thoracic department of a tertiary hospital for further management. On arrival, the patient showed clinical evidence of cardiac tamponade, which he developed within one month of his initial presentation, and hemodynamic compromise. He was tachypneic, and his parameters showed evidence of cardiorespiratory compromise. On reviewing the images, the opinion was that the left-sided collection was more of a low-density mass rather than fluid or hemothorax. As the patient was hemodynamically worsening, it was decided that he undergoes immediate surgery to drain without tissue diagnosis. A left posterolateral thoracotomy was performed. Upon entering the pleural space, it was filled with a very large mass of gelatinous consistency with a very weak membranous capsule holding it, shown in Figure 3. The mass extended around the esophagus and aorta and was not separable from them. It was excised, and the end result was R2 resection, as vital structures were involved. However, the left lung showed re-expansion, and the pressure on the diaphragm and mediastinum was relieved. Histopathological examination of the surgical tissues confirmed low-grade soft tissue sarcoma with lipogenic differentiation. Pan CT of the patient ruled out any other site involvement, and thus the diagnosis of primary pleural myxoid liposarcoma was made. The patient had an uneventful postoperative recovery.

Adjuvant treatment modalities were considered as the patient had incomplete tumor resection. Complete resection was not considered as the tumor was invading vital mediastinal structures. Radiotherapy (XRT) was also not feasible because of the large radiation field and anticipated toxicity of mediastinal structures. Thus, the patient was given chemotherapy. At mid-cycle of chemotherapy, a new CT scan was performed, which showed rapid progression of the disease. The findings were explained to the patient, and he understood the severity of his condition. The treatment from then onwards was palliative to improve the quality of life (QOL). He passed away four months later.

## Discussion

About 24% of all pleural effusions are caused by malignant illness [6]. The most common malignant tumors of the pleura are metastases [7]. Only 10% of pleural tumors are primary malignant neoplasms, with mesothelioma being the most frequent (90%). Primary pleural lymphoma and various sarcomas, including liposarcoma, are rare malignancies that can emerge from the pleura [8,9]. PPLs are extremely rare pathologies. The exact incidence is not known. PPL is more prevalent in males between 19 and 80 years, according to Carrillo et al. [10]. Furthermore, because of a delayed growing process that involves displacement and compression of mediastinal structures, the symptoms of PPL are not specific [10]. The most common presenting complaints are chest pain, cough, and dyspnea [8].

Even though there are various minimally invasive techniques for histologic identification of mediastinal tumors and cysts, open surgical access is occasionally required. Traditional methods for diagnosing mediastinal tumors include mediastinoscopy, thoracoscopy, and exploratory thoracotomy. These operations require general anesthesia and hospitalization [11].

PPLs with myxoid and well-differentiated subtypes had a five-year survival rate of 71%, compared to 12.5% for the other subtypes in Wong et al.'s series [9]. For PPL with no radiologic evidence of local invasion of the important surrounding structures, complete surgical excision with radiotherapy is the treatment of choice [9]. Palliative chemotherapy and radiotherapy are frequently used to treat unresectable tumors [12].

The currently available literature shows that survival varies between seven months and eight years. Wong et al. published a similar report to our case with the patient alive five months following resection and adjuvant treatment [9]. In our case, the presentation was very advanced, so the survival was very poor.

## Conclusions

Myxoid pleural liposarcomas are very rare entities. They are slow growing with an expansile rather than infiltrative behavior. They may present late with compression symptoms on adjacent structures, as in our case. There is no common consensus on the treatment options for such cancer, but complete surgical resection remains the mainstay for better outcomes.
